# Effects of siRNA on RET/PTC3 Junction Oncogene in Papillary Thyroid Carcinoma: From Molecular and Cellular Studies to Preclinical Investigations

**DOI:** 10.1371/journal.pone.0095964

**Published:** 2014-04-23

**Authors:** Hafiz Muhammad Ali, Giorgia Urbinati, Hubert Chapuis, Didier DesmaEle, Jean-Rémi Bertrand, Patrick Couvreur, Liliane Massaad-Massade

**Affiliations:** 1 Université Paris-Sud 11, Laboratoire de Vectorologie et Thérapeutiques Anticancéreuses, UMR 8203, Villejuif, France; 2 CNRS, Villejuif, Laboratoire de Vectorologie et Thérapeutiques Anticancéreuses, UMR 8203, Villejuif, France; 3 Gustave Roussy, Laboratoire de Vectorologie et Thérapeutiques Anticancéreuses, UMR 8203, Villejuif, France; 4 Institut Galien, UMR CNRS 8612, Université Paris-Sud 11, Faculté de pharmacie, Châtenay-Malabry, France; Aligarh Muslim University, India

## Abstract

*RET/PTC3* junction oncogene is typical of radiation-induced childhood papillary thyroid carcinoma (PTC) with a short latency period. Since, *RET/PTC3* is only present in the tumour cells, thus represents an interesting target for specific therapy by small interfering RNA (siRNA). Our aim is to demonstrate *in vitro* and *in vivo* molecular and cellular effects of siRNA on *RET/PTC3* knockdown for therapeutic application.First, we established a novel cell line stably expressing *RET/PTC3* junction oncogene, named RP3 which was found tumorigenic in nude mice compared to NIH/3T3 mouse fibroblasts. Among four siRNAs and five concentrations tested against *RET/PTC3*, an efficient siRNA *RET/PTC3* and an appropriate dose (50 nM) were selected which showed significant inhibition (*p*<0.001) of gene (RT-qPCR) and protein (Western blot) expressions. This siRNA was found efficient in RP3 cells (harbouring *RET/PTC3*) but non-efficient in BHP10-3 SCmice cell line (harbouring RET/PTC1) showing that a specific siRNA against fusion sequence is required to target the junction oncogene. *In vitro* siRNA *RET/PTC3* showed significant (*p*<0.001) inhibitory effects on RP3 cell viability (MTT assay) and on invasion/migration (IncuCyte scratch test) with blockage of cell cycle at G0/G1 phase (flow cytometry) and induced apoptosis by caspase-3 and PARP1 cleavage (WB). After intravenous injection in nude mice, respective squalene (SQ) nanoparticles (NPs) of siRNA *RET/PTC3* significantly (*p*<0.001) reduced RP3 tumour growth, oncogene and oncoprotein expressions, induced apoptosis and partially restored differentiation (decrease in Ki67). Hence, our findings highly support the use of siRNA *RET/PTC3*-SQ NPs as a new promising treatment for patients affected by PTC expressing *RET/PTC3*.

## Introduction

Papillary thyroid carcinoma (PTC) accounts for about 80% of thyroid malignancies [1], [2] and ionizing radiations are described as an important etiological factor for PTC development [3]. Indeed, thousands of people developed thyroid cancer after Chernobyl catastrophe [4], [5]. PTC is characterized by gene rearrangements affecting *RET* (rearranged during transfection) proto-oncogene, located on chromosome 10q11.2 and coding for a cell membrane tyrosine kinase receptor [6]. This gene plays a role in the regulation of cell survival, growth, differentiation and migration [7]. In PTC, *RET* fuses with different ubiquitous genes located on same or alternate chromosomes to give various *RET/PTC* fusion rearrangements; leading to an abnormal expression of a chimeric RET protein that is constitutively activated in thyroid follicular cells [8]. Among the 13 fusion patterns of RET with 12 different genes reported so far [9], *RET/PTC1* and *RET/PTC3* are the major variants, while the others are very rare and of little clinical significance. *RET/PTC1* results from the fusion of *RET* with *H4* gene (*CCDC6/D10S170*) while *RET/PTC3* arises from *RET* fusion with *ELE1* gene (also designated as nuclear receptor co-activator 4*; NCOA4*, *RFG or ARA70* [10]). The spatial proximity of *RET* gene with *CCDC6* (10q21) or *ELE1* (10q11.2) during thyrocyte interphase explains the *RET/PTC1* or *RET/PTC3* formation [11]. *RET/PTC3* has been found to be more frequent than *RET/PTC1* in cases of thyroid cancers exposed to post-Chernobyl radiations, mostly found in young subjects [12], [13], [14] and is usually associated with an aggressive phenotype, a short latency period and poor prognosis [9].

Current thyroid cancer therapy includes total thyroidectomy and functional lymph node dissection, followed by radioiodine therapy and suppression of serum thyroid-stimulating hormone. This approach is usually successful in early stage disease, however, treatment options for advanced PTC malignancy remain unsatisfactory and the prognosis is also poor [15]. Thus, new alternative treatments are an utmost need. Because of the strong involvement of *RET/PTC3* fusion oncogene in tumour development, gene inhibition therapy specifically targeting *RET/PTC3* would be an alternative and personalised therapy for PTC harbouring this junction oncogene. Thus, in order to conceive more effective and specific treatment, we developed a new gene oriented therapy by siRNA to target *RET/PTC3* rearrangement, which is only present in the tumour cells and not in the surrounding normal cells.

However, no cellular model was available until now to investigate more deeply the molecular targets and the effects of this fusion oncogene. Establishing a cellular model, stably expressing *RET/PTC3* rearrangement was fundamental to assess tumoral properties linked to *RET/PTC3* expression such as cellular invasiveness, migration and proliferation ability and most importantly, to develop new therapies. Although, specific gene inhibition property of siRNA is well documented and already exploited in clinical investigations, many hurdles need to be overcome to achieve specific and effective gene knockdown *in vitro* and *in vivo* [16]. Indeed, design of an appropriate sequence is crucial in the choice of siRNA for therapeutic purposes because off-targeting phenomenon due to silencing of gene sharing sequence homologies need to be avoided. Moreover, specific gene inhibition must be strong and durable enough to allow modulation of events strictly triggered by the oncogene impairment such as cellular proliferation and invasion. In addition, siRNA needs efficient drug delivery systems *in vivo,* to avoid degradation and to achieve its entry and accumulation in the desired tissue especially whilst systemic administration is required.

So far, a wide variety of approaches including viral vectors and non-viral delivery systems have been employed to deliver siRNA in vivo [17], [18], however, the safety of these vectors is questionable [16]. Therefore, we recently conceived a new strategy to deliver siRNAs, based on their conjugation to squalene (SQ); a natural and nonionic biocompatible lipid [19], [20], [21]. This concept termed “squalenoylation” has been first developed for the delivery of anti-cancer [22] and antiviral nucleosides analogues [23] and has been recently enlarged to other molecules [24].

The aim of this study was to achieve an efficient therapy against *RET/PTC3* junction oncogene using siRNA technology. We first established *RET/PTC3* expressing cell line, named RP3, from murine NIH/3T3 fibroblasts. Then siRNAs were designed and studied for the *RET/PTC3* silencing effects, apoptosis and cell cycle regulation pathways. The most efficient siRNA RET/PTC3 was conjugated to squalene and the efficiency of the resulting nanoassemblies (siRNA RET/PTC3-SQ NPs) was validated *in vitro* before testing in preclinical experiments. Our results strongly suggest that siRNA RET/PTC3-SQ NPs could be accredited as a potential new pharmacological approach for papillary thyroid carcinoma with *RET/PTC3* junction oncogene.

## Materials and Methods

### Chemicals

Squalene, DMSO (dimethyl sulfoxide), *in vitro* LDH toxicology assay kit (TOX7), MTT (3-(4,5-dimethylthiazol-2-yl)-2,5-diphenyl tetrazolium bromide) reagent, *cis*-diammine platinum(II) dichloride (cisplatin), puromycin and paraformaldehyde (PFA, 16%) were purchased from Sigma-Aldrich Chemical Co. (Saint Quentin Fallavier, France). Dulbecco's modified Eagle medium (DMEM), Opti-MEM, new born (NBCS) and fetal (FCS) calf serums, Lipofectamine 2000 (1 mg/ml), Annexin-V-Fluos with propidium iodide (PI) kit and PCR primers were purchased from Life technologies (Saint Aubin, France). BD Matrigel (Basement Membrane Matrix Growth Factor Reduced) was purchased from BD Bioscience (Le Pont de Claix, France).

### Cell lines and cell culture transformation protocol

Mouse embryo fibroblasts NIH/3T3 (ATCC CRL-1658 Manassas, USA) were grown in DMEM medium supplemented with 10% NBCS, penicillin (100 U/ml) and streptomycin (10 µg/ml) and maintained at 37°C in an atmosphere of 5% CO_2_ and 95% humidity. RET/PTC3 cDNA cloned in a pBABE-puro plasmid under the LTR promoter, was kindly provided by Dr A. Fusco (University Federico II, Naples, Italy) [10]. To obtain the stable cell line expressing *RET/PTC3* oncogene, NIH/3T3 cells were seeded in Petri dishes and transfected with RET/PTC3 cDNA pBABE-puro plasmid with Lipofectamine 2000 as recommended by the supplier. After 24 h incubation, medium was replaced with DMEM medium supplemented with NBCS and containing 5 mg/ml puromycin (as selecting agent). RP3 colonies were collected and expanded in the same growth medium containing selecting agent. All selected colonies were verified for expression of *RET/PTC3* by RT-PCR followed by agarose gel electrophoresis (Fig. S1A).

The human BHP 10-3 SC_mice_ cell line is a variant of human BHP 10-3 cells, harboring *RET/PTC1* fusion oncogene rearrangement, colonizes in soft agar and has tumorigenic properties. It was kindly provided by Dr. G. Clayman (MD Anderson Cancer Center, Houston, USA (MT2010-6894) [25]. Cells were grown in DMEM medium supplemented with 10% FCS, penicillin (100 U/ml) and streptomycin (10 µg/ml) and maintained at 37°C in an atmosphere of 5% CO_2_ and 95% humidity.

### Oligonucleotides design and synthesis

The *RET/PTC3* mRNA sequence (Fig. S2A) was obtained by blasting *ELE1*/*RET* TK (human papillary thyroid carcinoma PC-9, mRNA, GI: 547383) with Human *RET* proto-oncogene mRNA (GI: 38274) and Homo sapiens nuclear ELE1 mRNA sequence (GI: 296286). *RET/PTC3* oncogene expression in RP3 cells was verified by RT-PCR using three primers sets to amplify *ELE1* part, *RET* part and *RET/PTC3* junction sequence in collected RP3 cell clones (primers are represented in colors and underlined in Fig. S2).

### Characterization of RP3 cells

The transformed RP3 cells (stably expressing *RET/PTC3* oncogene) were compared to original NIH/3T3 cell line for: i) morphological features by using a phase contrast microscope and ii) growth rate by IncuCyte system (Essen Instruments, Ann Arbor, MI, USA); which allows an automated and non-invasive method of monitoring live cells in culture. Cells were seeded at different concentrations (2.0, 3.0, 4.0 or 5.0×10^3^ cells/well) in 96-well plate. Every four hours, each well was scanned by IncuCyte and growth rate was calculated. Proliferation curve was drawn by dividing cell confluence value at each time point by the initial confluence rate. Results are the mean of three independent experiments.

### siRNA RET/PTC3 design and synthesis

Four siRNAs were designed against *RET/PTC3*, according to Reynolds et al., 2004 [26] and named as siRNA RET/PTC3 #1, #2, #3 and #4 (Table S1). The siRNA scrambled sequence was used as control (siRNA CT). A siRNA previously designed against *RET/PTC1* junction oncogene by Gilbert-Sirieix *et al*. was used to assess the specificity of the designed siRNA RET/PTC3 sequence [27]. All single stranded siRNAs were chemically synthesized by Eurogentec France SASU (Angers, France) as 21-mer with two 3′ overhanging 2′-deoxynucleotide residues, as described by Tuschl [28]. A 3-mercaptopropyl phosphate group was introduced at the 3′-end of siRNA sense strand to allow squalene bio-conjugation. Double stranded siRNAs were generated as previously described and duplex formation was assessed by 4% agarose gel electrophoresis [20].

### Synthesis, preparation and characterization of siRNA RET/PTC3-SQ NPs

The bio-conjugate of siRNA RET/PTC3-SQ was synthesized by Michael addition of 3′-thiol group with squalene maleimide and the corresponding nanoparticles were prepared by nano-precipitation as previously published [19], [20], [21]. The hydrodynamic diameter (nm) and the Zeta potential (mV) were measured by laser light scattering using a Zetasizer 4 (Malvern Instrument Ltd, Orsay, France).

### *In vitro* cell transfection

In order to: i) assess the most efficient siRNA RET/PTC3 designed (siRNA #1, #2, #3 or #4), ii) found the most efficient siRNA concentration, iii) analyse the gene knockdown efficiency of siRNA and iv) study the *RET/PTC3* knockdown efficiency after siRNA RET/PTC3 squalenoylation, transient transfections were performed in RP3 cells using Lipofectamine 2000 as previously described [27]. Moreover, BHP10-3 SC_mice_ cells were transfected with both siRNA RET/PTC1 or siRNA RET/PTC3 to test the specificity of siRNA in gene inhibition.

Briefly, 6 µl (1 µg/µl) of Lipofectamine 2000 were mixed with siRNA in serum free OPTI-MEM culture medium (2 ml) in 6-wells plates. After 4 h of incubation, the OPTI-MEM medium was replaced by complete culture medium supplemented with serum then incubated at 37°C in 5% CO_2_ for 24 h, 48 h and 72 h. For the choice of an efficient siRNA concentration, RP3 cells were transfected with the selected most efficient siRNA RET/PTC3 at 10 nM, 25 nM, 50 nM and 100 nM concentrations and incubated for 24 h. Nanoparticles (siRNA RET/PTC3-SQ NPs or siRNA CT-SQ NPs) were added at 50 nM concentration, either alone or in the presence of Lipofectamine 2000, as described above. At the end of the treatments, mRNA and proteins were extracted from the cells to be analysed for gene and protein knockdown.

### Reverse Transcription-quantitative PCR (RT-qPCR)

Total RNA extraction and RT-qPCR were performed as previously described [19], [21]. For determination of *RET/PTC3* gene expression, following primers were used: *RET/PTC3:* 5′-TTCAGCGAATGGCTCCTT-3′ (forward); 5′-CCGTTGCCTTGACCACTT-3′ (reverse); *18S*: 5′-GTAACCCGTTGAACCCCATT-3′ (forward) and 5′-CCATCCAATCGGTAGTAGCG-3′ (reverse). *RET/PTC1* and *RPL13A* primers were previously described [27]. The amplification was monitored with StepOnePlus PCR System (AB Applied Biosystems, Villebon-sur-Yvette, France) using GoTaq qPCR Master Mix (Promega, Charbonnieres Les Bains, France) according to manufacturer's instructions. Samples were run in triplicate, relative abundance of each target was normalized to 18S expression in RP3 cell line or to RPL13A in BHP10-3 SC_mice_ cell line and gene regulation was determined by the quantitation-comparative ΔΔC_T_ method [29].

### Immunoblotting

Cells were lysed and total proteins were extracted, then loaded on 10% polyacrylamide gel (NuPAGE Bis Tris Mini Gels 10%, Life technologies, Saint-Aubin, France) as previously described [19], [21]. Primary antibodies specific for RET (Rabbit Ret EPR2871; 1∶1000, abcam Biochemicals Paris, France, for BHP10-3 cell line or Rabbit Ret sc-167; 1∶200, Santa Cruz Biotechnology Heidelberg, Germany, for RP3 cell line), GAPDH (mouse Abcys, 1∶1000; Sigma-Aldrich, used as an internal control), Caspase-3 (Rabbit Caspase-3 antibody, 1∶1000, Cell Signalling technology, Saint Quentin Yvelines, France) and PARP-1 [Anti-PARP-1 (Ab-2) Mouse mAb (C-2-10), 1∶200, Calbiochem EMD Millipore, Darmstadt, Germany] were used. Blots were then washed and incubated with corresponding secondary anti-rabbit or anti-mouse antibodies conjugated to HRP (Horseradish peroxidase, 1∶3000, Cell Signalling technology). Quantification of relative protein expression was done as previously described and results are expressed compared to non-treated cells [19], [21].

### Viability assay

The viability of RP3 cells was evaluated after 24 h, 48 h or 72 h incubation with 50 nM of siRNA RET/PTC3, siRNA RET/PTC1 or siRNA CT by MTT method [20]. All experiments were set up in triplicate to determine means ± SD. Results are expressed in viability percentage of treated cells compared to non-treated cells.

### Measurement of LDH release

RP3 cells were seeded in 6 wells culture plates (0.5×10^6^ cells/well/2 ml medium) and transfected with 50 nM of siRNA RET/PTC3, siRNA RET/PTC1 or siRNA CT. The LDH cytotoxicity detection kit was used to measure cellular LDH release at 24 h, 48 h or 72 h post-transfection as described by the manufacturer instructions. Absorbance was then measured at 490 nm with a microplate reader (MRXII photometer, Thermo Scientific, Courtaboeuf, France). The ratio of released LDH over total LDH was calculated and presented as relative LDH release compared to non-treated cells.

### Cell invasion and migration assay

Scratch test was performed to assess the effects of siRNA on cell invasion and migration as previously described [27], [30]. Briefly, BD Matrigel was plated in 96-well ImageLock cell migration plates (Essen Bioscience, Michigan, USA) at 24 h before seeding of RP3 cells. Cells (2000/well) were then plated and once reached 90% of confluence, were transfected with 50 nM of siRNA RET/PTC3, siRNA RET/PTC1 or siRNA CT. Cells monolayer was then scratched with a 96-pin WoundMaker (Essen Bioscience, Michigan, USA) and cells were covered either with Matrigel (8.5 mg/ml in DMEM medium) for invasion or fresh DMEM medium for migration assays and maintained in culture until complete wound confluence (40 h for invasion assay or 85 h for migration assay). Cell mobility was monitored by Incucyte at every 4 h using “scratch wound” scan type selection.

### Cell cycle analysis by flow cytometry

RP3 cells were transfected with 50 nM of siRNA RET/PTC3, siRNA RET/PTC1 or siRNA CT as described above. After 24 h, 48 h or 72 h, cells were collected and incubated in DNA staining buffer (50 µg/mL PI in 0.1% sodium citrate, 0.1% Triton 100X, 100 µg/ml RNase A, pH 7.8) in the dark for 30 min at room temperature. The samples were then analyzed by flow cytometer (Accuri C6 Flow Cytometer, BD Biosciences, San Jose, USA) and results are presented as cell percentage distributed in each phase of cell cycle in treated compared to non-treated cells.

### Annexin-V apoptosis assay

Apoptosis was determined by flow cytometry analysis using Annexin-V kit. RP3 cells were transfected with 50 nM of siRNA RET/PTC3, siRNA RET/PTC1 or siRNA CT. Cisplatin (100 µM) was used as a positive control for apoptosis. After 24 h, 48 h or 72 h incubations, medium and cells were collected and centrifuged, then stained with Annexin-V kit according to manufacturer's instructions. Experiments were performed in triplicate and data represent apoptotic cells percentage compared to non-treated cells.

### Animal studies

All animal experiments and use of NIH/3T3 and RP3 cell lines were approved by the institutional Ethics Committee of Animal Experimentation (CEEA) and research council (Integrated Research Cancer Institute in Villejuif; IRCIV) registered in the French Ministry of Higher Education and Research (Ministère de l'Enseignement supérieur et de la Recherche; MESR) under the authorization number CEEA IRCIV/IGR n°26: 94–226, n°: 2011-09. They were carried out according to French laws and regulations under the conditions established by the European Community (Directive 2010/63/UE). Administration of nanoparticles was done under isoflurane anesthesia, animals were sacrificed by CO_2_ inhalation before tumour collection and all efforts were made to minimize animal suffering. Six-weeks old female nude *nu/nu* mice were purchased from Institute Gustave Roussy's animal facility and housed in a sterilised laminar flow caging system with food and water *ad libitum*.

### Tumorigenicity of NIH/3T3 and RP3 cells

Both NIH/3T3 and RP3 cells were injected sub-cutaneously at three different concentrations (0.5, 1.0 or 2.0×10^6^ cells/mouse) into the flank of nude mice (n = 5/group) in 100 µl PBS. Tumour growth was followed during 8 days and tumours were collected for *RET/PTC3* gene expression analysis by RT-PCR followed by agarose gel electrophoresis.

### *In vivo* efficiency of siRNA RET/PTC3-SQ nanoparticles

RP3 cells were sub-cutaneously (*s.c.*) inoculated (0.5×10^6^ cells/mouse in 100 µl PBS). When tumours reached about 50 mm^3^, mice (n = 5/group) were treated intra-venously (*i.v.*) either with saline solution (NaCl 0.9%) or siRNA RET/PTC3-SQ NPs, non-vectorized siRNA RET/PTC3, siRNA CT-SQ NPs dispersed in 100 µl of 0.9% NaCl solution at the rate of 0.5 mg/kg/injection (cumulative dose  = 2.5 mg/kg/mouse). Mice were monitored daily for tumour growth and body weight and then sacrificed at the end of the experiment (day-17). Tumours were immediately collected in liquid nitrogen for RT-qPCR analysis and Western blotting and in PFA (4%) for immuno-histo-chemistry (IHC) studies.

### RNA and protein extractions from tumours

Tumours were grinded, total RNA and protein were extracted, as previously described. RT-qPCR and Western blot were performed to assess respectively *RET/PTC3* mRNA and RET/PTC3 protein expressions as well as cleavage of caspase-3 and PARP-1. Results are presented as relative mRNA or protein expressions compared to tumours treated with 0.9% NaCl solution.

### Immunohistochemistry

Tumour tissues were embedded in paraffin and 4 µm thick sections were prepared and stained with hematoxilin-eosin-saffranin (HES). For IHC detection of proliferation, samples were incubated with a monoclonal rabbit anti-Ki67 antibody (Lab Vision/Neomarkers, Fremont, USA, 1∶200) followed by Rabbit PowerVision Kit (UltraVision Technologies, North Andover, USA). The signals were revealed with chemiluminescence DAB PowerVision kit (ImmunoVisionTechnologies Co., Hillsborough, USA). Sections were examined with Zeiss Axiophot microscope (Microscopy and Imaging center, Texas, USA) and digitized using a Nikon slide scanner 8000 (Nikon, Melville, USA). Quantification of Ki67 stained cells was achieved using Adobe Photoshop 6 (Adobe, Paris, France) and the ratio between stained and unstained cells was established.

### Statistical analysis

All data are presented as mean ± SD (Standard deviation). One-way analysis of variance (ANOVA) was employed to compare multiple treatments. Using a linear regression model, doubling time was calculated by GraphPad Prism 4 software. While all pair wise comparisons between different treatment groups were done by least significant difference (LSD) Post-hoc test by using “InVivoStat” software. *p<*0.05 was considered as statistically significant level.

## Results

### Establishment of a cellular model RP3 expressing *RET/PTC3* junction oncogene

We succeeded to establish a cell line (called RP3) stably expressing *RET/PTC3* junction oncogene. This was testified by the presence of *RET/PTC3* amplified fragment in all collected clones either by using primers that cover 114 bp of *ELE1* part and 91 bp of *RET* (205 bp) (Fig. S1A) or by using specific primers against the *ELE1* part (173 bp) or the *RET* part (235 bp) (Fig. S2B).

The doubling time of RP3 cells was found significantly higher than that of wild-type NIH/3T3 (*p*<0.05), respectively 33±2 h *vs* 28±1 h (Fig. S1B). This is probably the result of morphological modifications due to introduction of *RET/PTC3* fusion oncogene. In fact, RP3 cells are round in shape with long filipodes while NIH/3T3 cells are star shaped and devoid of filipodes. Moreover, the RP3 cells form characteristic button cell clumps in culture while the wild-type NIH/3T3 cells grow in typical monolayer (Fig. S1C).

### siRNA selection and *in vitro* gene and protein silencing efficiency

**Figure 1:**
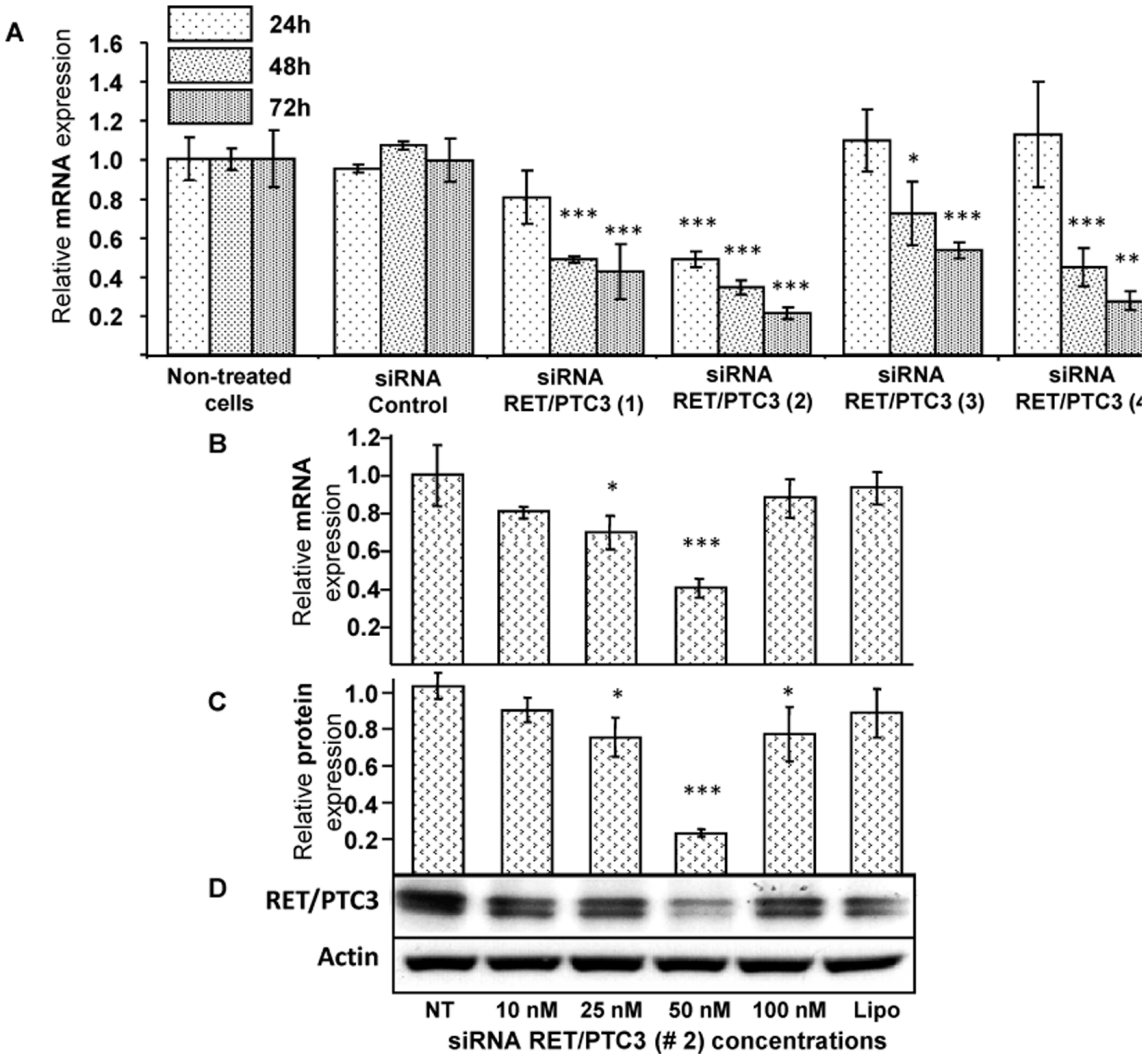
Selection of an efficient siRNA RET/PTC3 and its effective dose. **A**. Four siRNAs RET/PTC3 along with siRNA Control (for sequences see: Table S1 and Fig. S2) were transfected at 50 nM with Lipofectamine 2000 in RP3 cell line. After 24 h, 48 h and 72 h post-transfection, RNA was extracted and the *RET/PTC3* mRNA expression was analyzed by RT-qPCR and recorded as relative mRNA expression of treated compared to non-treated (NT) cells. **B, C and D**. siRNA RET/PTC3 #2 was transfected with Lipofectamine (Lipo) at 10 nM, 25 nM, 50 nM and 100 nM concentrations in RP3 cells. After 24 h post-transfection, RNA and protein were extracted and *RET/PTC3* mRNA and protein expressions were respectively analyzed by RT-qPCR (**B**) and Western blot (**C, D**). Quantification of Western blot was performed by scanning films with Gel Doc XR+ Systems images and analysing results by Bio-Rad Image Lab software scanning (Bio-Rad Laboratories). The results are expressed as relative RET protein expression compared to non-treated cells. All data were analysed by one-way ANOVA followed by LSD post-hoc test. Stars represent the significant difference between the treatment groups compared to non-treated cells. * = *p*<0.05, *** = *p*<0.001. NT = non-treated cells, Lipo = Lipofectamine 2000.

The *RET/PTC3* gene silencing efficiency of the designed siRNAs was tested by RT-qPCR at 24 h, 48 h and 72 h post-transfection incubations. All four siRNAs were found efficient in gene silencing but siRNA RET/PTC3 #2 was selected for future experiments (will be called later as siRNA RET/PTC3) since it significantly decreased *RET/PTC3* mRNA expression (*p<*0.001) (Fig. 1A).

Among the various concentrations (10 nM, 25 nM, 50 nM and 100 nM) tested of siRNA RET/PTC3 #2 at 24 h post-transfection incubation, 50 nM concentration showed significant (*p<*0.001) gene (Fig. 1B) and protein (Fig. 1C, 1D) inhibitions and was thus selected for further experiments.

### Specificity and long-term gene silencing efficiency of siRNA RET/PTC3

**Figure 2:**
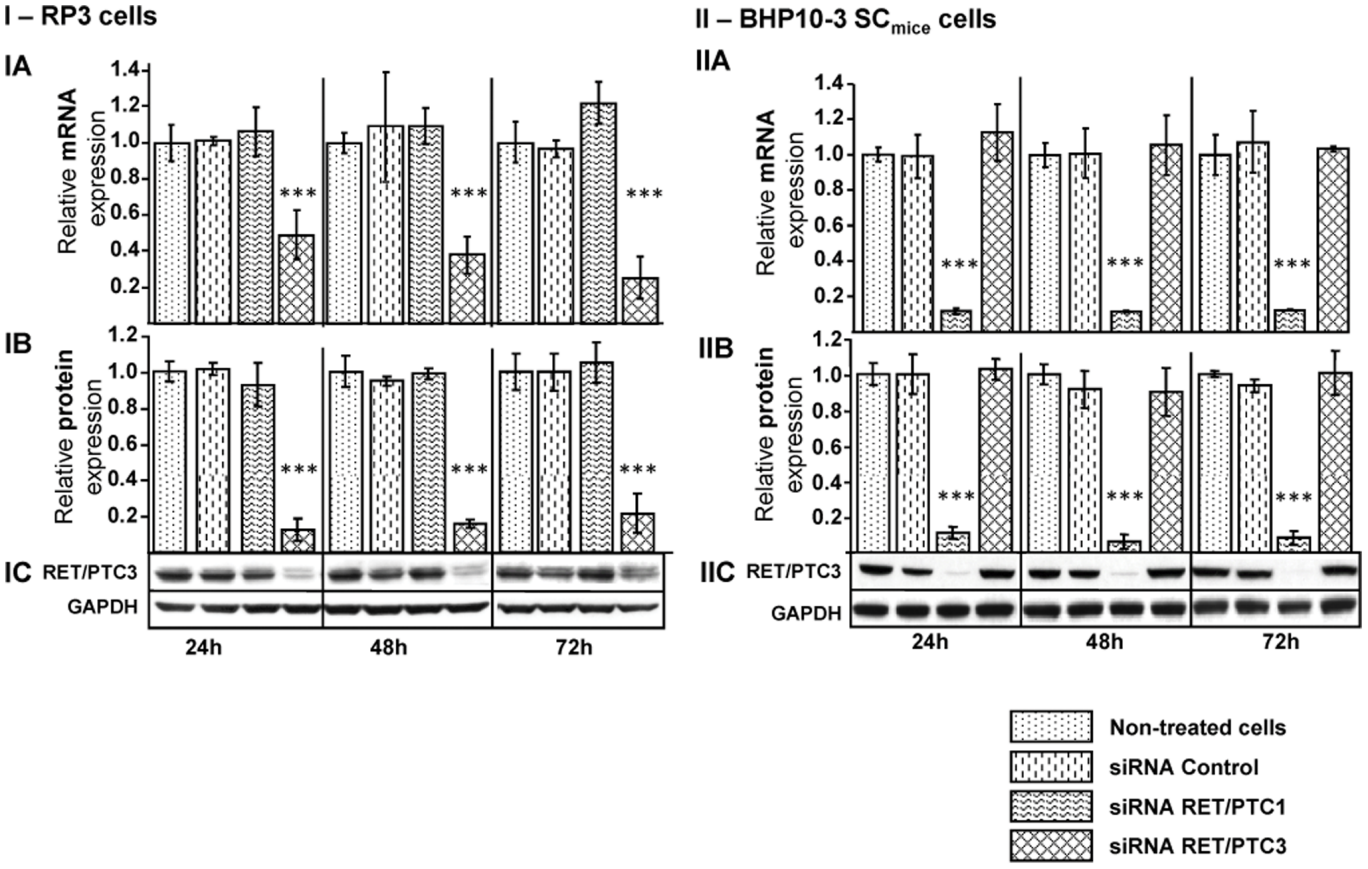
Specificity of siRNA RET/PTC3 for inhibition of RET/PTC3 fusion oncogene expression in RP3 (I) and in BHP10-3 SCmice (II) cells. siRNAs (RET/PTC3, RET/PTC1 or Control) were transfected at 50 nM concentration in RP3 (**I**) and BHP10-3 cells (**II**). After 24 h, 48 h and 72 h post-transfection, RT-qPCR (**IA, IIA**) and Western blot (**IC, IIC**) were performed for relative mRNA and protein expressions. Quantification of relative RET protein expression (IB, IIB) was performed by Bio-Rad Image Lab software scanning, results are expressed as relative RET protein expression compared to non-treated cells. Data were analysed by one-way ANOVA followed by LSD Post-hoc test. Stars represent the significant difference between the treatment groups compared to non-treated cells. *** = *p*<0.001.

By RT-qPCR, we found that siRNA RET/PTC3 significantly reduced (*p<*0.001) *RET/PTC3* mRNA level in RP3 cells compared to both siRNA CT and siRNA RET/PTC1 (Fig. 2, IA, bars 4, 8 and 12). The down-regulation of *RET/PTC3* mRNA level by siRNA RET/PTC3 was paralleled with a decrease of RET/PTC3 protein content in Western blot (Fig. 2, IB and IC; lines 4, 8 and 12). Worth of notice, when the cells were transfected with siRNA RET/PTC3, corresponding gene and protein inhibitions were observed up to 72 h, suggesting the long-term inhibitory effects of siRNA (Figure 2, left panel, RP3 cells).

Specificity was tested in the *RET/PTC1*-expressing human BHP10-3 SC_mice_ cell line (Figure 2, right panel). Treatment with siRNA RET/PTC3 did not affect either the *RET/PTC1* mRNA or protein expressions in BHP10-3 SC_mice_ cell line (Figure 2, IIA IIB and IIC, bars 4, 8 and 12). In contrast, the siRNA RET/PTC1 decreased (*p<*0.001) *RET/PTC1* oncogene and oncoprotein expressions in BHP10-3 SC_mice_ cell line (Figure 2, IIA, IIB and IIC; bars 3, 7 and 11).

### Effects of siRNA RET/PTC3 on cell viability, toxicity, invasion and migration

**Figure 3:**
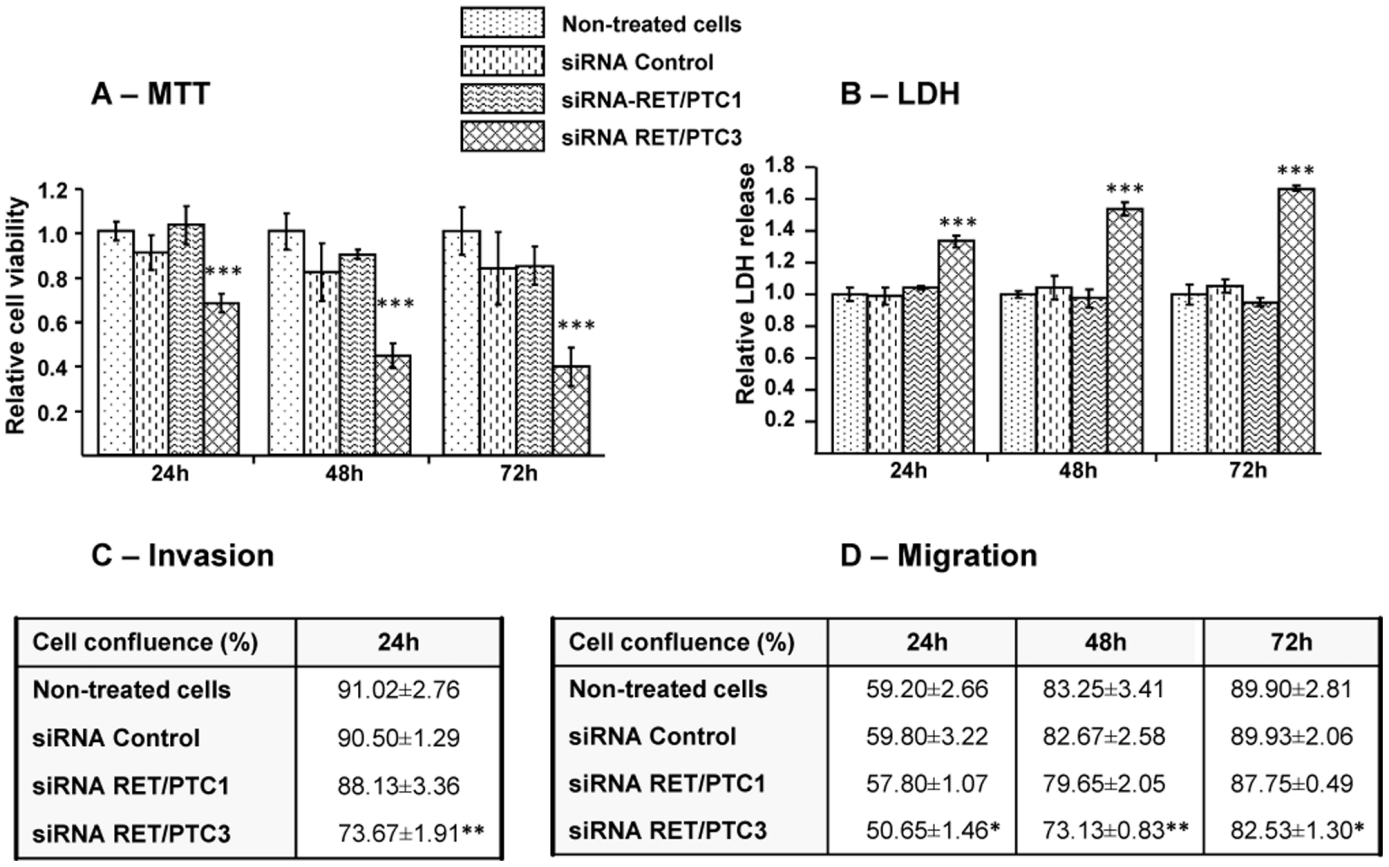
Inhibition of cell growth, invasion and migration by siRNA RET/PTC3. siRNA RET/PTC3 was tested for cell viability (**A**), LDH release (**B**), invasion (**C**) and migration (**D**) in RP3 cells. siRNAs (RET/PTC3, RET/PTC1 or Control) were transfected with Lipofectamine at 50 nM concentration in RP3 cells. (**A**) Cell viability was performed by MTT assay and (**B**) LDH release by LDH toxicology assay kit as described in Materials and Methods section. Measurements are performed at 24 h, 48 h and 72 h post-transfection. Monolayer RP3 cells was scratched and monitored every 4 h for invasion (**C**) or migration (**D**) assays by IncuCyte. Data were analysed by ANOVA followed by LSD Post-hoc test and presented as relative expressions in treated compared to non-treated cells. Stars represent the significant difference between the treatment groups compared to non-treated cells. * = *p*<0.05, ** = *p*<0.01, *** = *p*<0.001.

Effects of siRNA RET/PTC3 on RP3 cell viability were assessed by MTT and lactate dehydrogenase (LDH) cell toxicology assays. A significant inhibition (*p<*0.001) in growth rate (∼30% at 24 h and 60% at 48 h and 72 h) was observed in the cells treated with siRNA RET/PTC3 compared to non-treated cells (Fig. 3A). These results were further supported by LDH toxicology assay where a highly-significant LDH release was observed after similar treatment conditions, *p<*0.001 (Fig. 3B). Moreover, a significant inhibition of RP3 cell invasion (3C) and migration (3D) was observed under the effect of siRNA RET/PTC3 compared to non-treated cells or to other treatments (siRNA RET/PTC1 or siRNA CT). Since cells proliferate faster in Matrigel, the analysis for invasion assay was ended at 40 h as the wound reached the complete cell confluence.

### Effects of siRNA RET/PTC3 on RP3 cell cycle and death pathways

**Figure 4:**
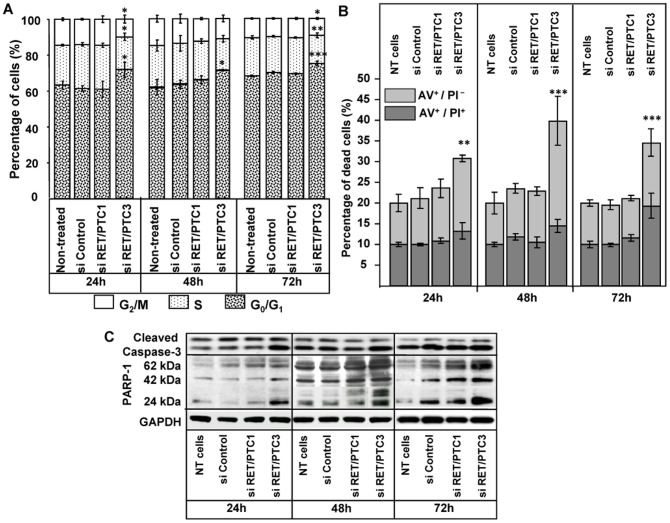
Effects of siRNA RET/PTC3 treatment on cell cycle and on cell death in RP3 cells. siRNAs (RET/PTC3, RET/PTC1 or Control) were transfected at 50 nM with Lipofectamine in RP3 cells and analysis were performed at 24 h, 48 h and 72 h post-transfection. A. Cells were incubated with PI and the repartition of cells within the different phases of cell cycle was analysed by FACS. B. Cells were incubated with with Annexin-V-Fluos (AV) and PI and analysed for early apoptosis (AV^+^/PI^−^) and late apoptosis/necrotic (AV^+^/PI^+^). Statistical analysis (ANOVA followed by LSD Post-hoc test) was performed to assess the difference between treatments.* = *p*<0.05; ** = *p*<0.01; *** = *p*<0.001; NT = non-treated; si = siRNA. **C**. Caspase-3 activation and PARP1 fragments were analyzed by Western blot, quantification of relative protein expressions was showed in Figure S4.

In order to assess the influence of siRNA RET/PTC3 on the repartition of cells within the cell cycle and its ability to induce cell death, flow cytometry analysis were performed in RP3 cells. A significant blockage of cell cycle in G_0_/G_1_ phase was observed at 24 h (*p<*0.05) and persisted up to 72 h; *p<*0.001 (Fig. 4A; bars 4, 8 and 12, Fig. S3). Annexine-V-Fluos analysis revealed an increased percentage of apoptotic RP3 cells after siRNA RET/PTC3 transfection at all time point incubations, however the most pronounced effect was found at 48 h, *p<*0.001 (Fig. 4B; bars 4, 8 and 12). Interestingly, at 72 h, an increase of PI staining was observed, suggesting a switch of early phases of apoptosis to late apoptosis/necrosis event (as depicted in Fig. 4B; bar 12). These observations were supported by cleavage of both caspase-3 and PARP1, as detected by Western blot (Fig. 4C; lines 4, 8 and 12, quantification in Fig. S4).

### *In vitro* gene and protein silencing efficiency of siRNA RET/PTC3-squalene nanoparticles

In order to protect the siRNA RET/PTC3 from degradation, the squalene (SQ) was coupled covalently to siRNA RET/PTC3 and siRNA CT. In water, both bio-conjugates self-assembled spontaneously into nanoparticles (NPs) of about 230 nm of diameter, with a poly-dispersity index of 0.2 and a ζ potential of −27 mV.

**Figure 5:**
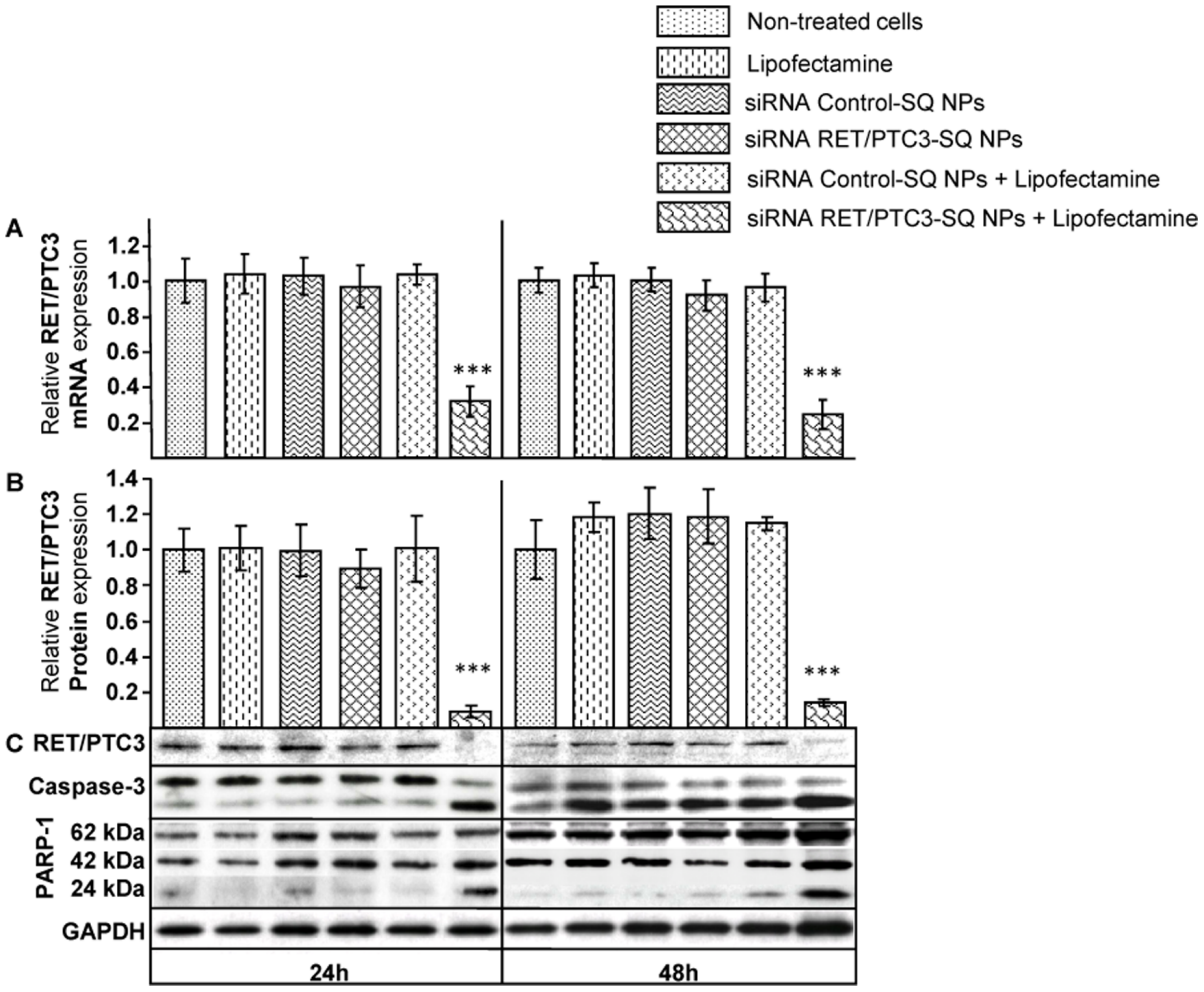
Vectorized siRNA RET/PTC3-SQ/Lipofectamine inhibits *RET/PTC3* oncogene and oncoprotein. RP3 cells were treated with siRNA RET/PTC3-SQ NPs and siRNA Control-SQ NPs at 50 nM with and without Lipofectamine 2000. **A**. After 24 h and 48 h post-transfection, RNA was extracted and RT-qPCR was performed to assess the relative RET/PTC3 mRNA expression compared to non-treated cells. A statistical analysis was performed to detect difference between treatments by using ANOVA followed by LSD Post-hoc test (*** = *p<*0.001). **B, C**. Proteins were extracted 24 h and 48 h after transfection and analyzed by Western blot using Ret, Caspase-3 and PARP-1 antibodies. GAPDH was used as loading control. Quantification of Caspase-3 and PARP-1 relative protein expressions is shown in Figure S5. NPs = Nanoparticles; SQ = Squalene.

Then, siRNA RET/PTC3-SQ NPs were tested for their ability to inhibit *RET/PTC3* oncogene and its corresponding oncoprotein in RP3 cells. The siRNA RET/PTC3-SQ NPs alone were unable to inhibit the RET/PTC3 mRNA and protein expressions (Fig. 5; bars and lines 4 for 24 h and 8 for 48 h) while when transfected with Lipofectamine 2000, a decrease of about 70% of *RET/PTC3* mRNA expression and a high reduction of protein content were observed (Fig. 5; bars and lines 6 for 24 h and 12 for 48 h). Moreover, cleaved caspase-3 and PARP1 were found to be highly increased (*p<*0.001) in the cells treated with siRNA RET/PTC3-SQ NPs once transfected with Lipofectamine 2000 compared to other groups (Fig. 5C and Fig. S5).

### *In vivo* studies: tumorigenicity of NIH/3T3 *vs* RP3 cells

In order to test the ability of transformed NIH/3T3 with *RET/PTC3* to give tumours, NIH/3T3 or RP3 cells were injected sub-cutaneously to nude mice at 0.5, 1.0 or 2.0×10^6^ cells/mouse. Unlike NIH/3T3 cells which were found non-tumorigenic, sub-cutenous xeno-grafted tumours were developed by all three injected RP3 cells concentrations (Fig. S6A). Tumours were collected at day-8 and *RET/PTC3* gene expression was verified by RT-PCR followed by agarose gel electrophoresis revealing persistent *RET/PTC3* junction oncogene expression in all collected tumours (Fig. S6B). To respect “the animal ethical laws” and because of the rapid tumour growth of RP3 cells, the 0.5×10^6^ cell/mouse injection was selected for further experiments.

### Efficacy of siRNA RET/PTC3-SQ NPs via intravenous route

**Figure 6:**
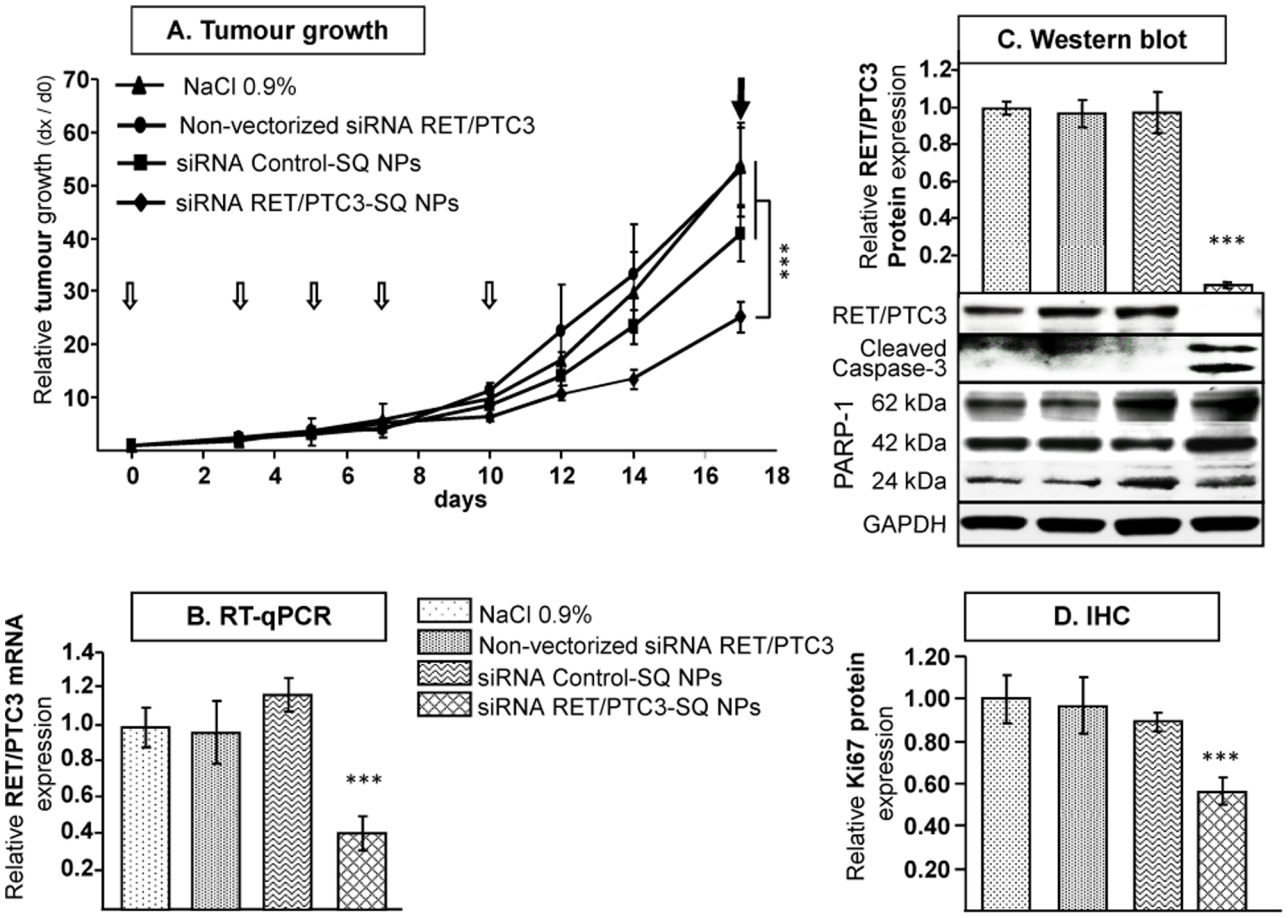
Vectorized siRNA RET/PTC3-SQ inhibit tumour growth and oncogene expression. **A**. nude mice (n = 5/group) were injected intravenously at 2.5 mg/kg cumulative dose (open arrows correspond to the days of treatment at 0, 3, 5, 7 and 10 days) either with NaCl 0.9%, non-vectorized siRNA RET/PTC3, siRNA Control-SQ NPs or siRNA RET/PTC3-SQ NPs. The tumour growth was followed during the course of experiment and mice were sacrificed and tumours were collected (filled arrow) at the end of the experiment (day-17). **B**. Relative *RET/PTC3* mRNA gene expression was performed by RT-qPCR. **C**. Proteins were extracted from tumours and analyzed by Western blot using Ret, Caspase-3 and PARP-1 antibodies. GAPDH was used as loading control. Quantification of relative RET/PTC3 protein expression is represented by bars (top right pannel) and those of Caspase-3 and PARP-1 is presented in Figure S7A and B. **D**. Quantification of immunohistochemistry (IHC) experiments carried out on untreated and treated tumours using Ki67 antibody, slides were analysed with a Zeiss microscope (Fig. S7C). To compare difference between treatments, a statistical analysis was performed by using ANOVA followed by LSD Post-hoc test (*** = *p<*0.001). NPs = Nanoparticles; SQ = Squalene.

The efficiency of siRNA RET/PTC3-SQ NPs was tested after *i.v.* injection in nude mice. When mice were treated with siRNA RET/PTC3-SQ NPs, tumour growth was strikingly inhibited compared to those receiving siRNA CT-SQ NPs, non-vectorized siRNA RET/PTC3 or saline solution (60% inhibition of tumoral volume compared to saline treated mice, LSD test, *p<*0.001) (Fig. 6A). However, neither weight loss, nor overall toxicity was observed in any group regardless treatments administered. At day-17, mice were sacrificed, tumours were collected and RT-qPCR, WB and IHC analysis were performed. *RET/PTC3* transcriptional products were found to be inhibited in tumours treated by siRNA RET/PTC3-SQ NPs (*p<*0.001) and these results were more significantly pronounced once we focused on RET/PTC3 protein expression in Western blot which was completely reduced (Fig. 6B and C). In addition, increasing expressions of cleaved caspase-3 and PARP1 were observed by Western blot (Fig. 6C and Fig. S7A and S7B). Ki67 proliferation marker was decreased in tumours treated with siRNA RET/PTC3-SQ NPs (Fig. 6D; bar 4 and Fig. S7C).

## Discussion

The RET/PTC3 rearrangement is associated with an aggressive form of PTC and was observed in the thyroid cancers at contaminated areas after Chernobyl explosion. Current treatments are often successful in early stage disease but remain unsatisfactory for advanced PTC. Thus, we aimed to conceive a new pharmacological approach targeting *RET/PTC3* oncogene by therapeutic siRNA. To achieve this goal, we initially established a cell line that expresses *RET/PTC3* because a cellular model is important for understanding the oncogenic transformation mechanisms and allows the development of new therapies. Moreover, to our knowledge there is no commercially available cell line that expresses this junction oncogene.

Interestingly, we found that the expression of the *RET/PTC3* junction oncogene was sufficient to transform the NIH/3T3 from immortal and not tumoral to tumoral cells. In fact, the transformed NIH/3T3 by *RET/PTC3* (RP3 cells) became spindle-shaped or round that is the consequence of cytoskeleton reorganization. Moreover, the RP3 cells are able to form tumour, once grafted in nude mice confirming the tumorigenic transformation of the immortalized NIH/3T3 cell line by *RET/PTC3* oncogene. Since *RET/PTC3* was found to be involved in tumour transformation, it can be considered as a potential target for a therapeutic approach.

In order to conceive more effective and specific treatment, we developed four siRNAs oriented within the junction sequence, to target only *RET/PTC3* rearrangement and proved their efficiency according to gene inhibition. The most efficient one in gene silencing in RP3 cells was found to be non-efficient in BHP10-3 SC_mice_ cell line (harbouring *RET/PTC1*). Moreover, siRNA RET/PTC1 was unable to inhibit *RET/PTC3* transcript in RP3 cells showing that a specific fusion sequence is required to target the junction oncogene. Taken together, these results showed that we successfully designed an efficient and specific siRNA capable of targeting the *RET/PTC3* sequence which is only expressed in tumour cells. Moreover, the long-term activity was confirmed by gene knockdown that suggests its use as therapeutic oligonucleotide.

In addition, the siRNA RET/PTC3 was found to significantly inhibit RP3 cell growth. Furthermore, a blockage of cells in G_0_/G_1_ phase was observed which may explain the delay in cell proliferation. Also, siRNA RET/PTC3 significantly increased the cell death by apoptosis and/or necrosis. Thus, it may have dual effects in RP3 cells; blocking cell proliferation and inducing cell death. This argument is further supported by LDH release in RP3 cells treated with siRNA RET/PTC3 reflecting toxicity induced by therapeutic siRNA.

In search of an underlying mechanism, protein expression analysis showed an increasing pattern of cleaved caspase-3 and PARP-1 fragments over time. Caspase-3 cleavage and PARP-1 activation demonstrate that siRNA RET/PTC3 might cause DNA damage which activates the cell death machinery for the destruction of tumour cells. It is considered that PARP-1 cleavage is a critical event and a switch point that directs death receptor signalling towards either apoptosis or necrosis. However, both pathways can also occur at the same time as described in L929 fibro-sarcoma cells where apoptosis and necrosis were concurrently induced by CD95 and TNF receptor activation [31]. It was also shown earlier, that *TRAIL* induces both apoptosis and cell death at acidic extra-cellular pH (pHe = 6.5) by caspase activation in HT29 human colon cancer cells [32]. Moreover, Wilson and Browning (2002) showed that *TNF* induces apoptosis and necrosis simultaneously in the HT29 adenocarcinoma cells [33]. We therefore postulate that the mechanism of cell death provoked by siRNA RET/PTC3 is a combination of both apoptosis and necrosis pathways which might be due to activation of death receptors by their respective ligands.

*In vitro* data suggested that the siRNA RET/PTC3 could have therapeutic effects if administrated to tumours carrying *RET/PTC3* junction oncogene. However, *in vivo* delivery of siRNA, is a key challenge because the biological efficacy of the siRNAs is hampered by their poor stability in biological fluids and low intracellular penetration due to their highly hydrophilic and anionic character [34]. In our previous research, we already used the squalenoylation approach to deliver siRNA RET/PTC1 [19], [20], [21]. Therefore, the acyclic isoprenoid chain of squalene was covalently coupled with siRNA RET/PTC3 at the 3′-terminus of the sense strand *via* maleimide-sulfhydryl chemistry [20]. Remarkably, the linkage of siRNA RET/PTC3 to squalene led to amphiphilic molecules that self-organised in H_2_O as siRNA RET/PTC3-SQ nanoparticles and were stable for several weeks at room temperature.

*In vitro*, these nanoparticles of siRNA RET/PTC3-SQ were failed to enter in to the RP3 cells without a cationic component but when these cells were transfected with squalenoyled siRNA RET/PTC3 in presence of Lipofectamine 2000, the NPs were able to inhibit RET/PTC3 oncogene and oncoprotein expressions and to activate cell death machinery. This also demonstrates that the siRNA RET/PTC3 is still active; after bio-conjugation with squalene, due to modification of the passenger sense strand only and the use of an ester hydrolysable bond between the squalene moiety and the siRNA part. *In vivo* on mice xenografted RP3 cells, siRNA RET/PTC3-SQ NPs were found to: i) inhibit tumour growth, ii) reduce RET/PTC3 oncogene and oncoprotein expressions, iii) induce cell death (cleavage of both caspase-3 and PARP-1) and iv) partially restore differentiation (decrease of Ki67 marker). The discrepancy between *in vitro* and *in vivo* nanoparticles uptake and silencing efficiency is probably due to differences between the enzymatic contents found *in vivo* biological fluids and in culture conditions, the possible rationalization was discussed previously [19], [21].

In conclusion, the significance of siRNA RET/PTC3-SQ NPs have been demonstrated in preclinical studies for thyroid cancer therapy and further pharmacological and clinical investigations can be proceeded to set-in the remedy of thyroid carcinoma. Furthermore, in future, we desire to extend our investigations to other cancer pathologies carrying junction oncogene, so that the “squalenoylation” could be used as a generic platform for the administration and the transport of therapeutic siRNA.

## Supporting Information

Figure S1 ***RET/PTC3*** **expression in the collected RP3 cell clones.** **A**. The *RET/PTC3* junction oncogene expression was verified in 12 selected RP3 cell clones and compared to wild-type NIH/3T3 cells. RT-PCR products were analysed by agarose gel electrophoresis. The expression of *RET/PTC3* was found at 205 bp in all clones. **B**. Evaluation of doubling-time in RP3 and NIH-3T3 cells. Every four hours, each well was scanned by IncuCyte™ and doubling time was calculated by linear regression model using GraphPad Prism 4 software. *** A statistical difference in cell growth was observed between RP3 and NIH-3T3 cells. Results represent the mean of three independent experiments. C. Morphology of RP3 and NIH/3T3 wild-type cell lines was observed by phase contrast microscope (×10 magnification).
(PDF)

Figure S2 **Sequence of RET/PTC3 fusion oncogene.** **A**. In colours, primers used to amplify the ELE1 Part **(**Blue), RET/PTC3 sequence (yellow) and RET part (green). Amplified fragments were designed in bold. In red, the most efficient siRNA designed to knockdown RET/PTC3. **B.** RT-PCR product were analysed by agarose gel electrophoresis in 3 randomly selected clones using the specific primers designed. As expected, the primers used amplified the corresponding sequence (173 bp for RET, 235 bp for ELE1 and 205 bp for RET/PTC3).
(PDF)

Figure S3 **siRNA RET/PTC3 induce blockage of RP3 cell cycle at G_0_/G_1_ phase.** RP3 cells were transfected with siRNA (RET/PTC3, RET/PTC1 and Control) at 50 nM with Lipofectamine. After 24 h, 48 h and 72 h post-transfection, cells were incubated with PI and analyzed by flow cytometer (Accuri C6 Flow Cytometer, BD Bioscience, USA). The area parameter histogram was used to determine the percentage of cells in G_0_/G_1_, S and G2-M phases. Data were analysed by one-way ANOVA followed by LSD Post-hoc test. Stars represent the significant difference between the treatment groups compared to non-treated cells. * = *p*<0.05, ** = *p*<0.01, *** = *p*<0.001.
(PDF)

Figure S4 **Induction of RP3 cell death by siRNA RET/PTC3 treatment. A and B**. Protein quantification of Caspase-3 (A) and PARP1 fragments (B) was done in treated cells by Bio-Rad Image Lab software scanning after western blot analysis and was presented as relative protein expression compared to non-treated cells. ANOVA followed by LSD Post-hoc test and *p<*0.05 were used to found statistical difference between treatments. * = *p*<0.05, ** = *p*<0.01, *** = *p*<0.001. NT = non-treated, Cl. = cleaved.
(PDF)

Figure S5 **Inhibition of** ***RET/PTC3*** **oncogene and oncoprotein by vectorized siRNA RET/PTC3-SQ/Lipofectamine.** Protein quantification of Caspase-3 (A) and PARP1 fragments (B) was done in treated cells by Bio-Rad Image Lab software scanning after western blot analysis and was presented as relative protein expression compared to non-treated cells. ANOVA followed by LSD Post-hoc test were used to found statistical difference between treatments, *** = *p*<0.001. NPs = Nanoparticles, SQ = Squalene, Cl. = cleaved.
(PDF)

Figure S6 **Tumorigenicity of RP3 cell line.** **A**. RP3 cells were injected sub-cutaneously at three different concentrations (0.5, 1.0 and 2.0×10^6^ cells/mouse) at the right flank of nude mice. The tumour growth was followed during the experiment and mice were sacrificed at day-8. **B**. A representative image of RT-PCR products analysed by agarose gel electrophoresis of 3 selected tumours showing the presence of RET/PTC3 oncogene at 205 bp in all tumours (1 = 0.5×10^6^ cells injected/mouse, 2 = 1.0×10^6^, cells injected/mouse, 3 = 2.0×10^6^ cells injected/mouse).
(PDF)

Figure S7 **Effect of vectorized siRNA RET/PTC3-SQ NPs on tumour growth, apoptosis and cell death. A and B.** Quantification of relative protein expression of cleaved Caspase-3 (**A**) and PARP1 fragments (**B**) in treated compared to non-treated cells. ANOVA followed by LSD Post-hoc test were used to found statistical difference between treatments, ** = *p*<0.01, *** = *p*<0.001. **C**. Immunohistochemical analysis revealed a decreased Ki67 positive nuclei only in the tumours treated with siRNA RET/PTC3-SQ NPs. Photograph magnification is 50X while inset 200X. NPs = Nanoparticles, SQ = Squalene.
(PDF)

Table S1 **Sequences of siRNAs designed against the RET/PTC3 junction oncogene.**
(PDF)
