# Supplementary figures and images for: Effects of siRNA on RET/PTC3 Junction Oncogene in Papillary Thyroid Carcinoma: From Molecular and Cellular Studies to Preclinical Investigations
Source: PLoS One. 2014 Apr 23;9(4):e95964. doi: 10.1371/journal.pone.0095964 (PMC3997558; doi:10.1371/journal.pone.0095964)

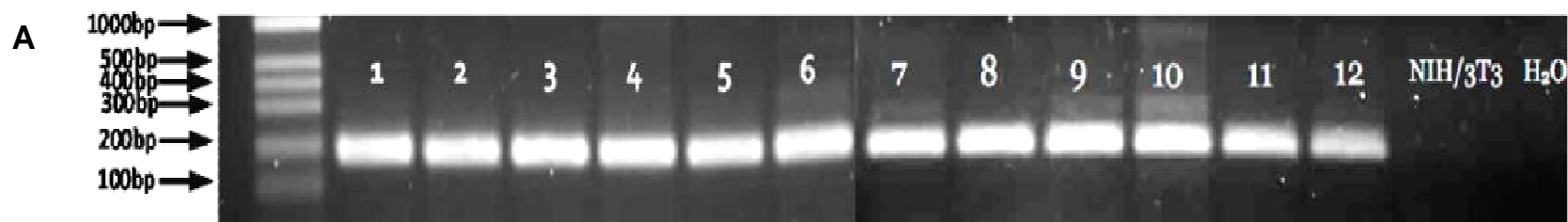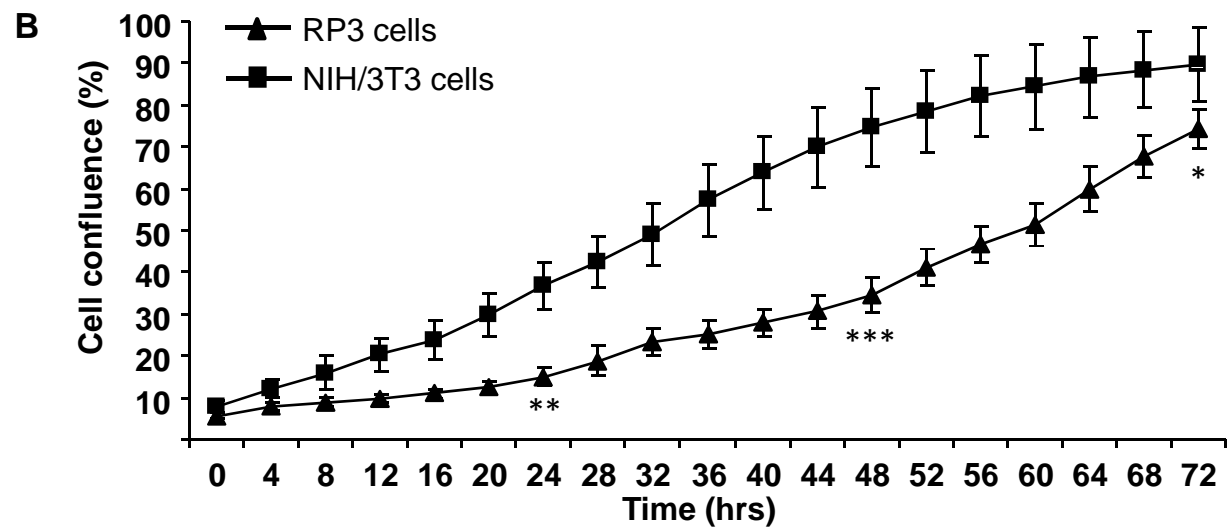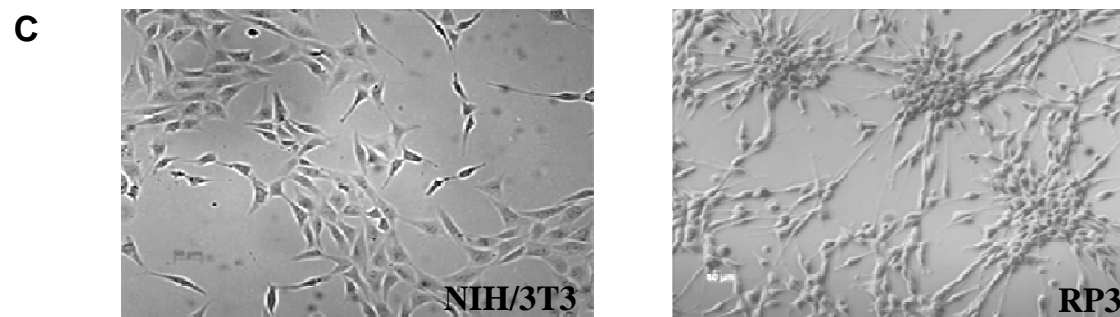

**Supplementary Figure S1.**

Supplement: Figure S1 — RET/PTC3 expression in the collected RP3 cell clones. A. The RET/PTC3 junction oncogene expression was verified in 12 selected RP3 cell clones and compared to wild-type NIH/3T3 cells. RT-PCR products were analysed by agarose gel electrophoresis. The expression of RET/PTC3 was found at 205 bp in all clones. B. Evaluation of doubling-time in RP3 and NIH-3T3 cells. Every four hours, each well was scanned by IncuCyte™ and doubling time was calculated by linear regression model using GraphPad Prism 4 software. *** A statistical difference in cell growth was observed between RP3 and NIH-3T3 cells. Results represent the mean of three independent experiments. C. Morphology of RP3 and NIH/3T3 wild-type cell lines was observed by phase contrast microscope (×10 magnification). (PDF) [file pone.0095964.s001.pdf]

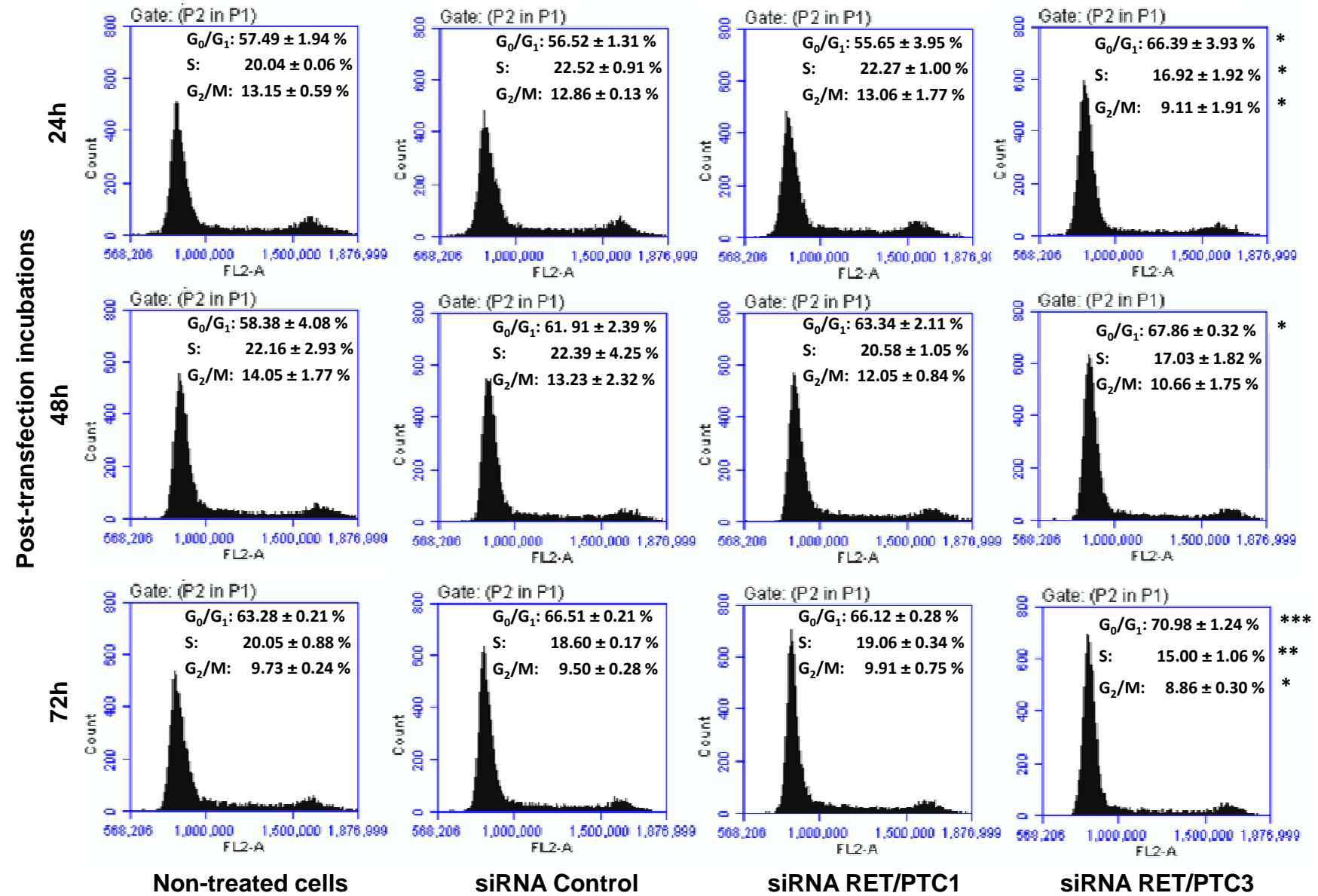

**Supplementary Figure S3.**

Supplement: Figure S3 — siRNA RET/PTC3 induce blockage of RP3 cell cycle at G0/G1 phase. RP3 cells were transfected with siRNA (RET/PTC3, RET/PTC1 and Control) at 50 nM with Lipofectamine. After 24 h, 48 h and 72 h post-transfection, cells were incubated with PI and analyzed by flow cytometer (Accuri C6 Flow Cytometer, BD Bioscience, USA). The area parameter histogram was used to determine the percentage of cells in G0/G1, S and G2-M phases. Data were analysed by one-way ANOVA followed by LSD Post-hoc test. Stars represent the significant difference between the treatment groups compared to non-treated cells. * = p<0.05, ** = p<0.01, *** = p<0.001. (PDF) [file pone.0095964.s003.pdf]

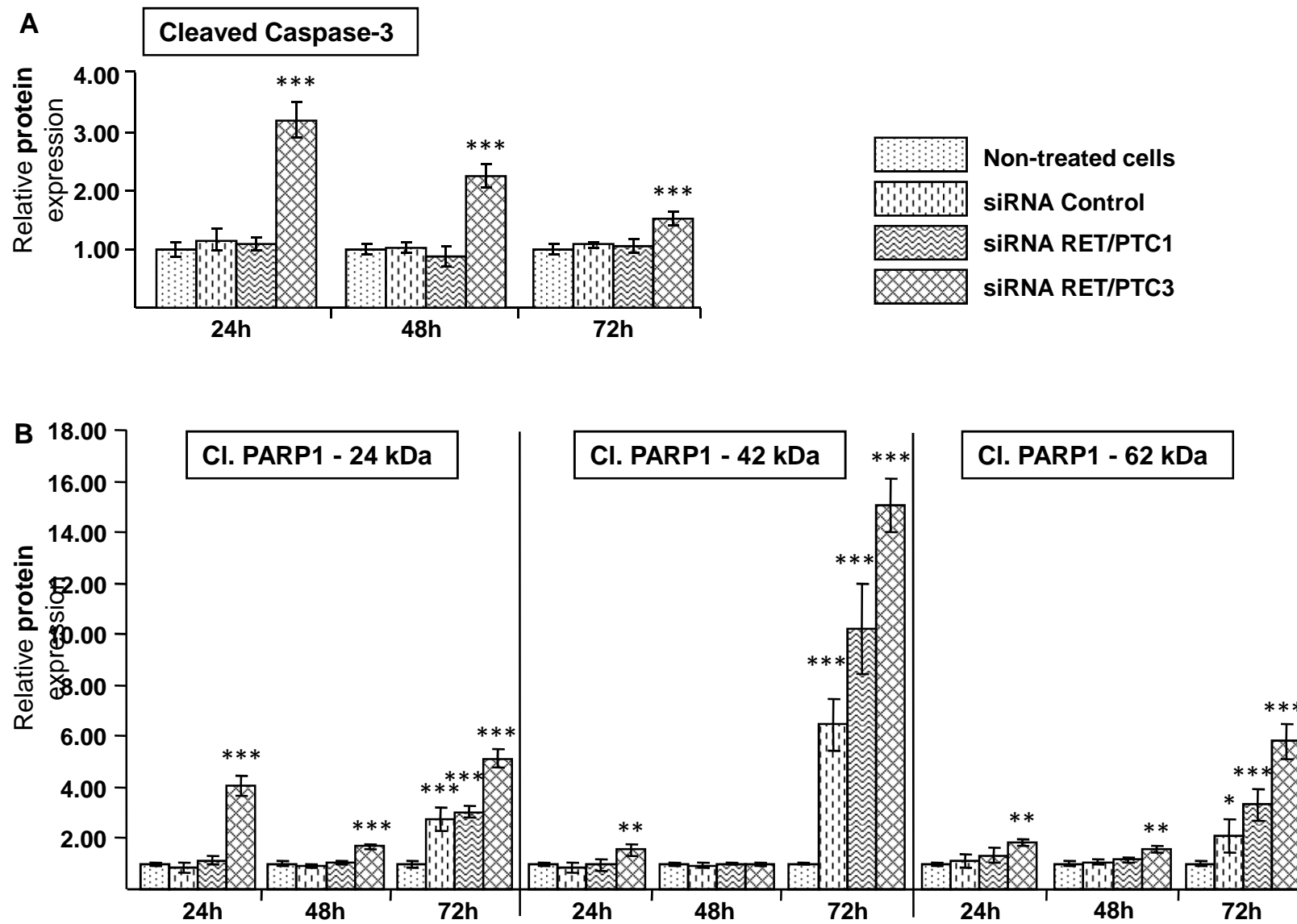

Supplementary Figure S4.

Supplement: Figure S4 — Induction of RP3 cell death by siRNA RET/PTC3 treatment. A and B. Protein quantification of Caspase-3 (A) and PARP1 fragments (B) was done in treated cells by Bio-Rad Image Lab software scanning after western blot analysis and was presented as relative protein expression compared to non-treated cells. ANOVA followed by LSD Post-hoc test and p<0.05 were used to found statistical difference between treatments. * = p<0.05, ** = p<0.01, *** = p<0.001. NT = non-treated, Cl. = cleaved. (PDF) [file pone.0095964.s004.pdf]

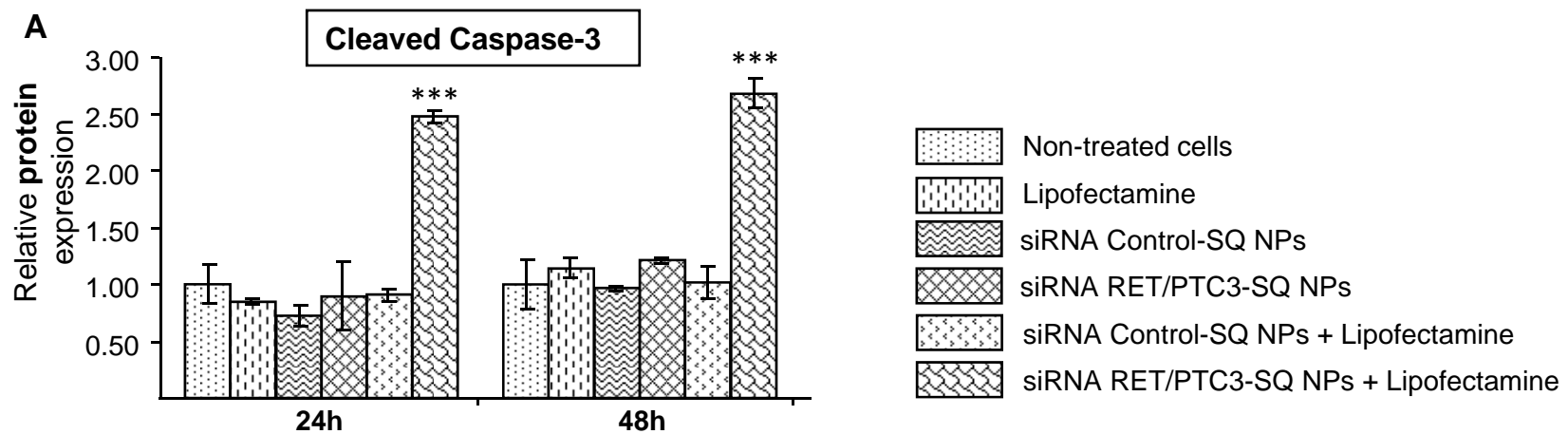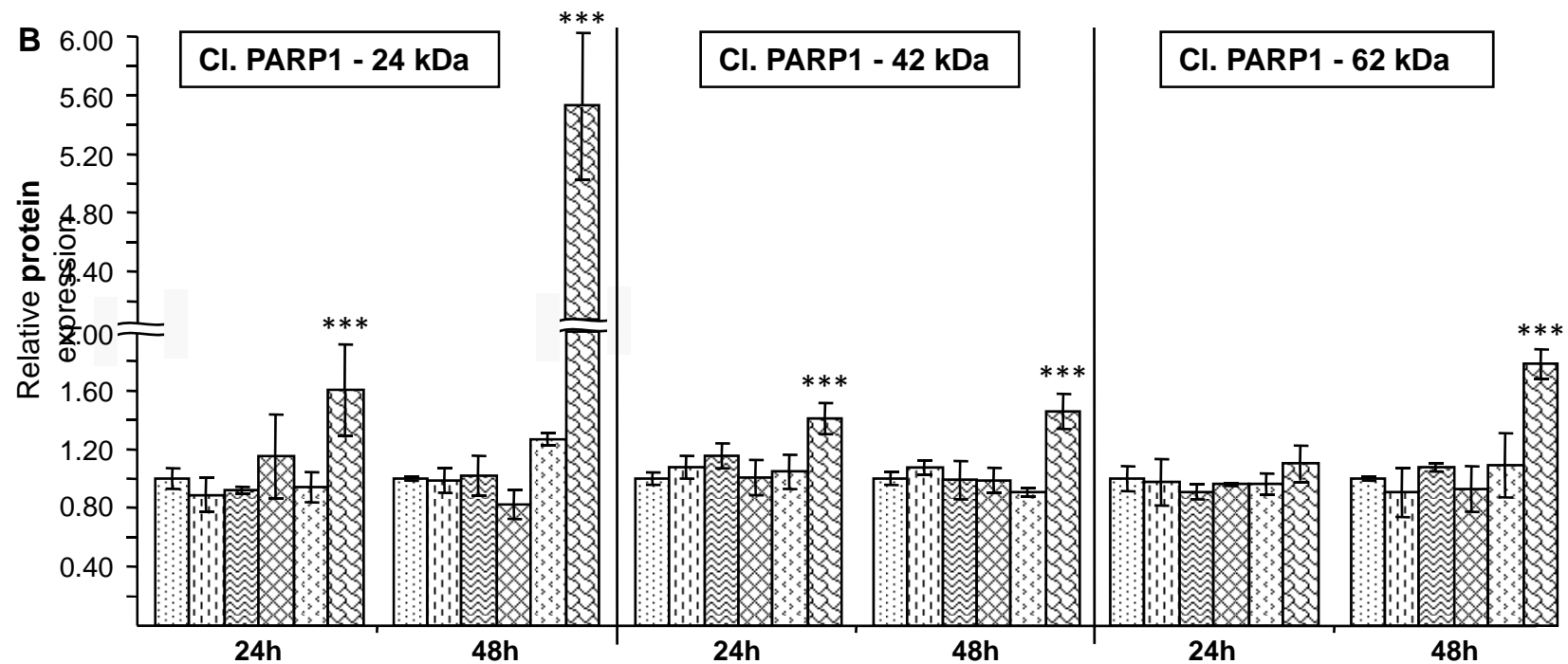

**Supplementary Figure S5.**

Supplement: Figure S5 — Inhibition of RET/PTC3 oncogene and oncoprotein by vectorized siRNA RET/PTC3-SQ/Lipofectamine. Protein quantification of Caspase-3 (A) and PARP1 fragments (B) was done in treated cells by Bio-Rad Image Lab software scanning after western blot analysis and was presented as relative protein expression compared to non-treated cells. ANOVA followed by LSD Post-hoc test were used to found statistical difference between treatments, *** = p<0.001. NPs = Nanoparticles, SQ = Squalene, Cl. = cleaved. (PDF) [file pone.0095964.s005.pdf]

**A**

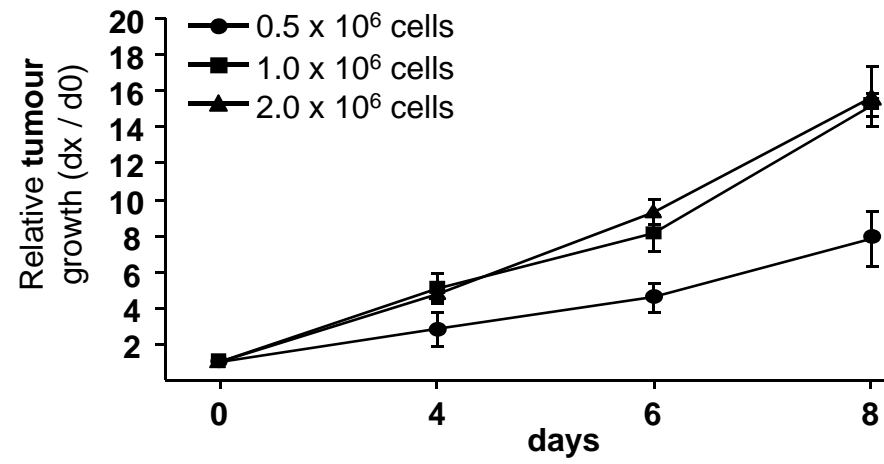

**B**

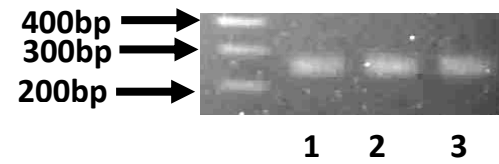

**Supplementary Figure S6.**

Supplement: Figure S6 — Tumorigenicity of RP3 cell line. A. RP3 cells were injected sub-cutaneously at three different concentrations (0.5, 1.0 and 2.0×10^6^ cells/mouse) at the right flank of nude mice. The tumour growth was followed during the experiment and mice were sacrificed at day-8. B. A representative image of RT-PCR products analysed by agarose gel electrophoresis of 3 selected tumours showing the presence of RET/PTC3 oncogene at 205 bp in all tumours (1 = 0.5×10^6^ cells injected/mouse, 2 = 1.0×10^6^, cells injected/mouse, 3 = 2.0×10^6^ cells injected/mouse). (PDF) [file pone.0095964.s006.pdf]

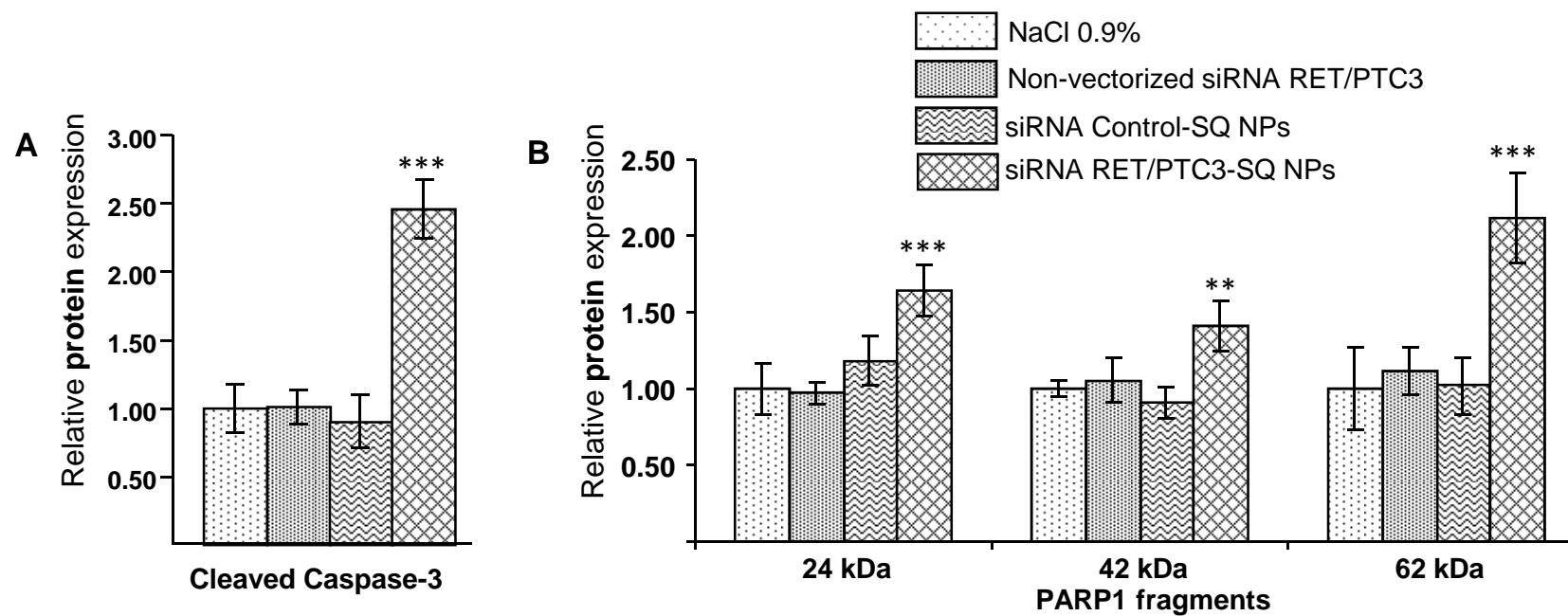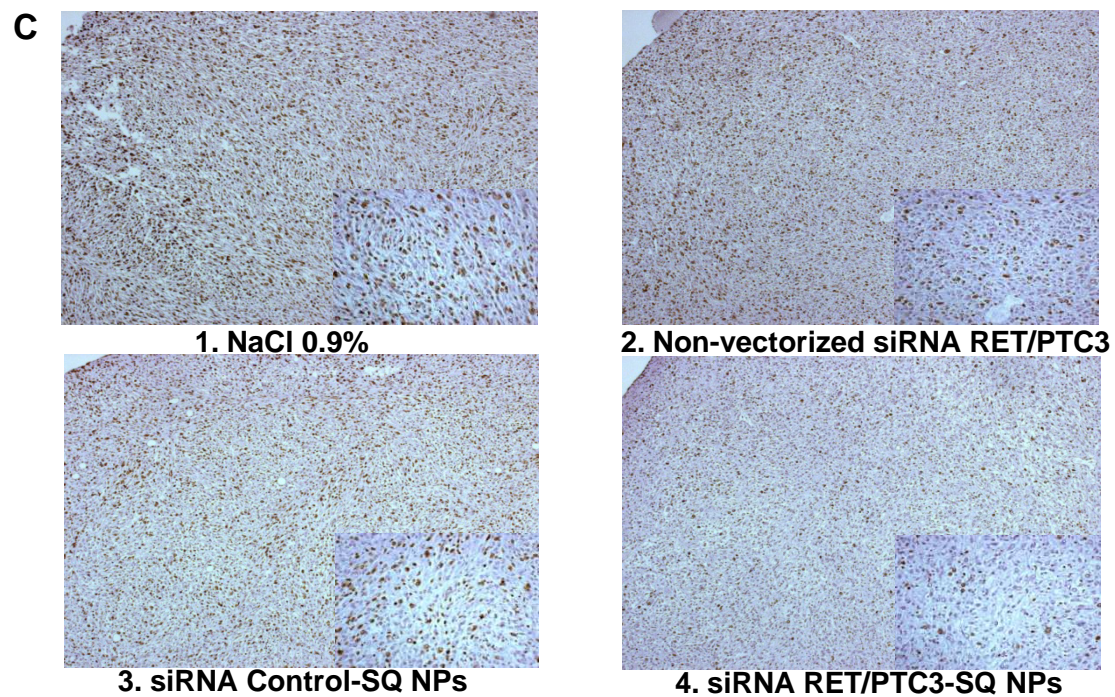

**Supplementary Figure S7.**

Supplement: Figure S7 — Effect of vectorized siRNA RET/PTC3-SQ NPs on tumour growth, apoptosis and cell death. A and B. Quantification of relative protein expression of cleaved Caspase-3 (A) and PARP1 fragments (B) in treated compared to non-treated cells. ANOVA followed by LSD Post-hoc test were used to found statistical difference between treatments, ** = p<0.01, *** = p<0.001. C. Immunohistochemical analysis revealed a decreased Ki67 positive nuclei only in the tumours treated with siRNA RET/PTC3-SQ NPs. Photograph magnification is 50X while inset 200X. NPs = Nanoparticles, SQ = Squalene. (PDF) [file pone.0095964.s007.pdf]
