# Supplementary material for: Effects of siRNA on RET/PTC3 Junction Oncogene in Papillary Thyroid Carcinoma: From Molecular and Cellular Studies to Preclinical Investigations
Source: PLoS One. 2014 Apr 23;9(4):e95964. doi: 10.1371/journal.pone.0095964 (PMC3997558; doi:10.1371/journal.pone.0095964)
Supplement: Figure S2 — Sequence of RET/PTC3 fusion oncogene. A. In colours, primers used to amplify the ELE1 Part (Blue), RET/PTC3 sequence (yellow) and RET part (green). Amplified fragments were designed in bold. In red, the most efficient siRNA designed to knockdown RET/PTC3. B. RT-PCR product were analysed by agarose gel electrophoresis in 3 randomly selected clones using the specific primers designed. As expected, the primers used amplified the corresponding sequence (173 bp for RET, 235 bp for ELE1 and 205 bp for RET/PTC3). (PDF) [file pone.0095964.s002.pdf]

A

ELE1 (NCOA4) part

5′AGAGGGCAGTCAAGGGCTTCTGGCTGACCCGAGCGGAGATCTCGCGAGACTGTCAGACGT ATGGCGAGAGGT
GTGGGGGAAGATTGTGTTGTCGCGAGAACTCTGCCTTTGGGCCGTAGGTTAGTGTGGGGCCGTG TCTCAGTCCA
CCCAAGGTCTCCTCGGATCGCCTGGAGAGGCACTCGGACCTGTT ATGTCTGGACACATTGCTTCAACATAGAAC
GCACATGAACAATGTGGAGGTCTAGGCTGGAATGGGGGCCCAGTTGACCACTTTTGCTCTAGCT GGAGCAGTGA
GGAGAATGAATACCTTCCAAGACCAGAGTGGCAGCTCCAGTAATAGAGAACCCCTTTTGAGGTG TAGTGATGCA
CGGAGGGACTTGGAGCTTGCTATTGGTGGAGTTCTCCCGGGCTGAACAGCAAATTAAAGATAAC TTGCGAGAGG
TCAAAAGCTCAGATTCACAGTTGCATAAGCCGTCACCTGGAATGT **TTAGAAGCCGTGAGGTATG GCTGTATGAA**
CAGGTGGACCTTATTTATCAGCTTAAAGAGGAGACACTTCAACAGCAGGCTCAGCAGCTCTACT CGTTATTGGG
CCAGTTCAATTGTCTTACTCATCAACTGGAGTGTACCCAAAACAAAGATCTAGCCAATCAAGTC TCTGTGTGCC
TGGAGAGACTGGGCAGTTTGACCCTTAAGCCTGAAGATT CAACTGTCCTGCTCTTTGA AGCTGACACAATTACT
CTGCGCCAGACCATCACCACATTTGGGTCTCTCAAAACCATTCAAATTCCTGAGCACTTGATGG CTCATGCTAG
TTCAGCAAATATTGGGCCCTTCCTGGAGAAGAGAGGCTGTATCTCCATGCCAGAGCAGAAGTCA GCATCCGGTA
TTGTAGCTGTCCCT **TTCAGCGAATGGCTCCTT GGAAGCAAACCTGCCAGTGGTTATCAAGCTCCTTACATACCC**
AGCACCGACCCCCAGGACTGGCTTACCCAAAAGCAGAC CTTGGAGAACAGTCAG|GAGGATCCAAAGTGGG AAT
TCCCTCGGAAGAACTTGGTTCTTGGAAAAACTCTAGGAGAAGGCGAATTTGGAA AAGTGGTCAAGGCAACGG CC
TTCCATCTGAAAGGCAGAGCAGGGTACACCACGGTGGCCGTGAAGATGCTGAAAGAGAACGCCT CCCCGAGTGA
GCTTCGAGACCTGCTGTCAGAGTTCAACGTCCTGAAGCAGGTCAACCACCCACATGTCATCAAA TTGTATGGGG
CCTGCAGCCAGGATGGCCCGCTCCTCCTCATCGTGGAGTACGCCAAATACGGCTCCCTGCGGGG CTTCCTCCGC
GAGAGCCGCAAAGTGGGGCCTGGCTACCTGGGCAGTGGAGGCAGCCGCAACTCCAGCTCCCTGG ACCACCCGGA
TGAGCGGGCCCTCACCATGGGCGACCTCATCTCATTTGCCTGGCAGATCTCACAGGGGATGCAG TATCTGGCCG
AGATGAAGCTCGTTCATCGGGACTTGGCAGCCAGAAACATCCTGGTAGCTGAGG **GGCGGAAGATGAAGATTTCG**
GATTTCGGCTTGTCCCGAGATGTTTATGAAGAGGATTCCTACGTGAAGAGGAGCCAGGGTCGGA TTCCAGTTAA
ATGGATGGCAATTGAATCCCTTTTTGATCATATCTACACCACGCAAAGTGATGTATGGT CTTTTGGTGTCCTGC
TGTGG GAGATCGTGACCCTAGGGGGAAACCCCTATCCTGGGATTCCTCCTGAGCGGCTCTTCAACCTTC TGAAG
ACCGGCCACCGGATGGAGAGGCCAGACAACTGCAGCGAGGAGATGTACCGCCTGATGCTGCAAT GCTGGAAGCA
GGAGCCGGACAAAAGGCCGGTGTTTGCGGACATCAGCAAAGACCTGGAGAAGATGATGGTTAAG AGGAGAGACT
ACTTGGACCTTGCGGCGTCCACTCCATCTGACTCCCTGATTTATGACGACGGCCTCTCAGAGGA GGAGACACCG
CTGGTGGACTGTAATAATGCCCCCCTCCCTCGAGCCCTCCCTTCCACATGGATTGAAAACAAAC TCTATGGCAT
GTCAGACCCGAACTGGCCTGGAGAGAGTCCTGTACCACTCACGAGAGCTGATGGCACTAACACT GGGTTTCCAA
GATATCCAAATGATAGTGTATATGCTAACTGGATGCTTTCACCCTCAGCGGCAAAATTAATGGA CACGTTTGAT
AGTTAACATTTCTTTGTGAAA (…)AAAAAAAAAAAAAAA3′

RET part

B

600bp
500bp
400bp
300bp
200bp
100bp

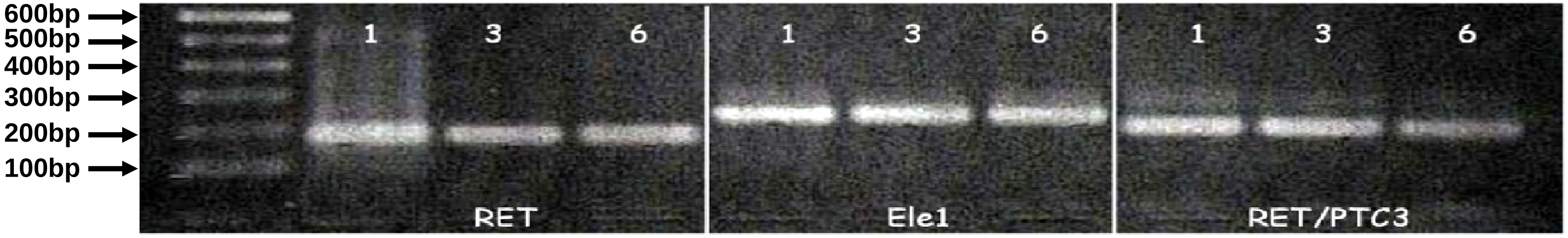

Supplementary Figure S2.
