# Supplementary material for: Effects of siRNA on RET/PTC3 Junction Oncogene in Papillary Thyroid Carcinoma: From Molecular and Cellular Studies to Preclinical Investigations
Source: PLoS One. 2014 Apr 23;9(4):e95964. doi: 10.1371/journal.pone.0095964 (PMC3997558; doi:10.1371/journal.pone.0095964)
Supplement: Table S1 — Sequences of siRNAs designed against the RET/PTC3 junction oncogene. (PDF) [file pone.0095964.s008.pdf]

Supplementary Table S1.

	**siRNAs designed**	**Nb GC**	**% GC**	**Stability**	**Nb bases *ELE1* (*NCOA4*)**	**Nb bases *RET***
**1**	5’ GAGAACAGUCAG**GAGGAUCCA** 3’<br>3’ ACCUCUUGUC**AGUCCUCCUAG** 5’	11	52 %	+ +	12	9
**2**	5’ AGAACAGUCAG**GAGGAUCCAA** 3’<br>3’ CCUCUUGUC**AGUCCUCCUAGG** 5’	10	48 %	+	12	10
**3**	5’ ACAGUCAG**GAGGAUCCAAAGU** 3’<br>3’ CUUGUC**AGUCCUCCUAGGUUU** 5’	10	48 %	+	8	13
**4**	5’ CAGUCAG**GAGGAUCCAAAGUG** 3’<br>3’ UUGUC**AGUCCUCCUAGGUUUC** 5’	11	52 %	+	7	14
**5**	5’ AAACUGCACAGAAGUACGGAG 3’<br>3’ UCUUUGACGUGUCUUCAUGCC 5’	siRNA Control (Scrambled sequence)				

Nb = Number

Supplementary Table S1.
